# Flavonoid and Leaf Gas Exchange Responses of *Centella asiatica* to Acute Gamma Irradiation and Carbon Dioxide Enrichment under Controlled Environment Conditions

**DOI:** 10.3390/molecules16118930

**Published:** 2011-10-25

**Authors:** Sina Siavash Moghaddam, Hawa Binti Jaafar, Maheran Abdul Aziz, Rusli Ibrahim, Asmah Bt Rahmat, Elizabeth Philip

**Affiliations:** 1 University Putra Malaysia, 43400 UPM Serdang, Selangor, Malaysia; E-Mails: drhawazej.postgrads@gmail.com (H.B.J.); maheran@agri.upm.edu.my (M.A.A.); asmah@medic.upm.edu.my (A.B.R.); 2 Agrotechnology & Biosciences Division, Malaysian Nuclear Agency, Bangi 43000 Kajang, Selangor, Malaysia; E-Mail: rusli_ibrahim@nuclearmalaysia.gov.my; 3 Forestry and Environment Division, Forest Research Institute Malaysia (FRIM), Kepong, 52109 Kuala Lumpur, Malaysia; E-Mail: philip@frim.gov.my

**Keywords:** *C.asiatica*, CO_2_ enrichment, gamma irradiation, leaf gas exchange

## Abstract

The study was couducted to investigate the effects of gamma irradiation and CO_2_ on flavonoid content and leaf gas exchange in *C.asiatica*. For flavonoid determination, the design was a split split plot based on Randomized Complete Block Design (RCBD). For other parameters, the designs were split plots. Statistical tests revealed significant differences in flavonoid contents of *Centella asiatica* leaves between different growth stages and various CO_2_ treatments. CO_2_ 400, G20 (400 = ambient CO_2_; G20 = Plants exposed to 20 Gy) showed 82.90% higher total flavonoid content (TFC) in the 5th week than CO_2_ 400 as control at its best harvest time (4th week). Increasing the concentration of CO_2_ from 400 to 800 μmol/mol had significant effects on TFC and harvesting time. In fact, 800 μmol/mol resulted in 171.1% and 66.62% increases in TFC for control and irradiated plants, respectively. Moreover, increasing CO_2_ concentration reduced the harvesting time to three and four weeks for control and irradiated plants, respectively. Enhancing CO_2_ to 800 µmol/mol resulted in a 193.30% (CO_2_ 800) increase in leaf biomass compared to 400 µmol/mol and 226.34% enhancement in irradiated plants (CO_2_ 800, G20) [800 = Ambient CO_2_; G20 = Plants exposed to 20 Gy] than CO_2_ 400, G20. In addition, the CO_2_ 800, G20 had the highest amount of flavonoid*biomass in the 4th week. The results of this study indicated that all elevated CO_2_ treatments had higher PN than the ambient ones. The findings showed that when CO_2_ level increased from 400 to 800 µmol/mol, stomatal conductance, leaf intercellular CO_2_ and transpiration rate had the tendency to decrease. However, water use efficiency increased in response to elevated CO_2_ concentration. Returning to the findings of this study, it is now possible to state that the proposed method (combined CO_2_ and gamma irradiation) has the potential to increase the product value by reducing the time to harvest, increasing the yield per unit area via boosting photosynthesis capacity, as well as increasing biochemicals (flavonoids) per gram DM.

## 1. Introduction

The daily intake of flavonoids in normal food, particularly fruits and vegetables, is 1–2 g. Modern physicians are increasing their use of pure flavonoids to treat many important common diseases due to their proven ability to restrain specific enzymes, to stimulate a number of hormones and neurotransmitters, and to scavenge free radicals [1]. Consumption of *Centella asiatica* as nutraceutical has shown to be a significant way to achieve the desired quantity of flavonoids from plant. However, flavonoids concentration in *Centella asiatica* is still relatively low. It is important to highlight that limited information exists on the potential and extent of enhanced production of secondary metabolites using physical elicitors, such as gamma radiation [2]. Moreover, few studies have documented the effects of ionizing radiation on photosynthesis. Therefore, it is important to examine the effects of ionizing radiation on the photosynthetic system.

In recent years, there has been an increasing interest in controlled environment (CE) plant production which reduces variation related to climate, soil, and nutrition [3,4,5], decreases contamination of samples by weeds, insects, and foreign matter [6] and enhances the standardization of secondary metabolite production [7]. As such, CE has the potential to boost the efficiency and quality of medicinal plants [8]. Additionally, a number of studies reported that CO_2_ enrichment enhances the production of secondary metabolites [9] and antioxidant activity [10].

Gamma radiation can interact with atoms and molecules to build up free radical levels in cells that cause modifications in important components of plant cells. It may induce remarkable morphological changes in plant tissues, as well as various biochemical responses at the cellular level. Papers by Kim and colleagues [11] have supported the hypothesis that gamma irradiation will induce growth stimulation by altering the hormonal signaling system in plant cells or increasing cells antioxidant activity to easily overcome daily environmental stress. Besides, gamma is able to alter physiological attributes to create new mutants with improved properties that can produce higher quantity of commercially essential metabolites and develop varieties that are agriculturally and economically significant, and contain high productivity potential [12,13,14].

Recently, researchers have shown an increased interest in evaluating the product of lipid peroxidation (malondialdehyde—MDA—as an indicator of free radicals) which is able to react with amino acid residues of membrane protein and nucleic acids, reduce membrane stability, and enhance membrane permeability. Therefore, cell structure and normal physiological function are destroyed [15]. CO_2_ enrichment, through provision of extra carbon to plants, is assumed to scavenge free radicals (decreasing MDA) through promotion of flavonoid biosynthesis.

The objectives of the following experiments in this study were to determine the effects of CO_2_ enrichment on the growth, leaf gas exchange and total flavonoid content in irradiated and non-irradiated accessions of *C.asiatica* and to identify mechanisms of CO_2_ enrichment and gamma irradiation effects on flavonoid concentration through MDA content of *Centella asiatica*.

It was hypothesised that CO_2_ would increase flavonoid compounds via excess carbon, as well as alleviate detrimental effects of gamma irradiation on photosynthesis apparatus and biomass. It is expected that CO_2_ and gamma irradiation would have synergistic effects to boost flavonoids in the leaves of *C.asiatica.*

## 2. Results and Discussion

### 2.1. Determination of Total Flavonoid Content and Leaf Biomass

Statistical tests revealed significant differences in flavonoid contents of *Centella asiatica* leaves between different growth stages and various CO_2_ treatments. The total flavonoid content (TFC) in the control (9.24 ± 0.028 mg/g DW) was found to be highest after four weeks of growth, and accordingly, this stands as the best time for leaf harvesting in the chamber without any treatment. When comparing the TFC of the control plants and the irradiated ones, results demonstrate that the irradiated plants exhibited significantly greater TFC in the 5th week (16.90 ± 0.021 mg/g DW) than the control plants at their best harvesting time (4th weeks). In other words, CO_2_ 400, G20 (400 = Ambient CO_2_; G20 = Plants exposed to 20 Gy) showed 82.90% higher TFC than CO_2_ 400 as control.

**Figure 1:**
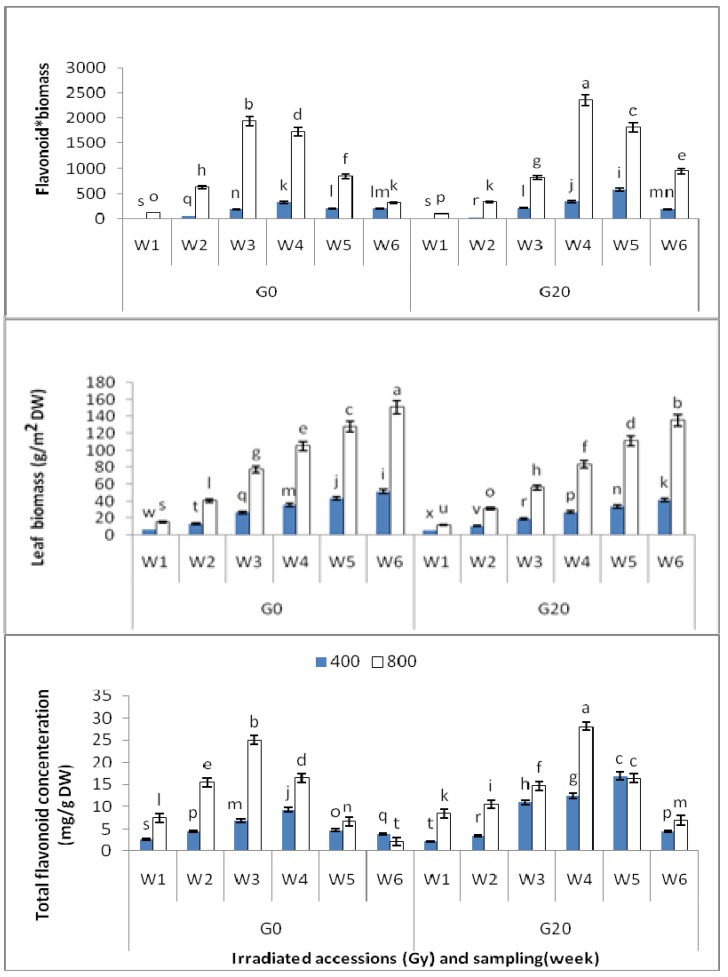
Interaction of different CO_2_ concentration, gamma dosage and leaf sampling on leaf total flavonoid, leaf biomass and flavonoid * leaf biomass in plants of *C.asiatica*; n = 2 (400 = Ambient CO_2_; 800 = 800 μmol/mol CO_2_ concentration; G0 = Control or non-irradiated plants; G20 = Plants exposed to 20 Gy; W = Weekly leaf sampling “for instance; W4 = 4th week after planting”).

Increasing the concentration of CO_2_ from 400 to 800 μmol/mol had significant effects on TFC and harvesting time. In fact, 800 μmol/mol resulted in 171.1% and 66.62% increases in TFC for control and irradiated plants, respectively. In other words, CO_2_ 800, G20 (800 = 800 μmol/mol CO_2_; G20 = Plants exposed to 20 Gy and CO_2_ 800 (800 μmol/mol CO_2_) had the two highest TFC values (28.16 ± 0.249 and 25.05 ± 0.277, respectively) compared to CO_2_ 400, G20 and CO_2_ 400 (400 μmol/mol CO_2_). Meanwhile, CO_2_ 800, G20 displayed 204.76% higher TFC than CO_2_ 400. Moreover, increasing CO_2_ concentration reduced the harvesting time to three and four weeks for control and irradiated plants, respectively (Figure 1).

In brief, according to the results obtained in the present study, it can be concluded that plants treated with gamma radiation and high concentration of CO_2_ displayed the highest total flavonoid content compared to other treatments. Moreover, the 4^th^ week of growth for irradiated plants exposed to high level of CO_2_ was found to possess the highest flavonoid content. 

Wang *et al.* [10] reported that strawberry fruit contains flavonoids with antioxidant properties, and CO_2_ enrichment conditions enhanced the phenolic compounds, flavonol, and anthocyanin concentrations. They further noted that plants grown under CO_2_ enrichment condition also had higher oxygen radical absorbance activity against [other types of oxygen] radicals in the fruit. For this reason, they concluded that atmospheric CO_2_ enrichment truly enhances plant secondary metabolite. 

Similar results were also observed in a number of vegetative tissues. For example, the growth enhancement associated with CO_2_ enrichment in CE was also accompanied via a boost in the total content of flavonoids in the vegetative tissues of *S. barhata and S. laterflora* [16]. More specifically, the total flavonoid concentration of *S. lateriflora* was significantly higher than *S. barbata* (1144 *vs.* 249 µg/g DW), and *S. lateriflora* hada more pronounced response to CO_2_ enrichment compared to *S. barbata.* The total flavonoid content increased by over 2.4 times at 1200 and 4.9 times at 3000 µmol/mol CO_2_ [16].

Previous studies have reported that sometimes, excess carbohydrates are used to enhance the biosynthesis of secondary carbon compounds in leaves. In the study by Estiarte *et al.* [17], for instance, leaves of spring wheat grown at 550 ppm CO_2_ exhibited 14% higher total flavanoid concentration than leaves exposed to 370 ppm CO_2_.

In general, there were significant differences among the different treatments on leaf biomass measurements. First, it was observed that increasing the concentration of CO_2_ from 400 to 800 µmol/mol had significant effects on the growth and development of *C.asiatica* leaves, such that enhancing CO_2_ to 800 µmol/mol (CO_2_ 800) resulted in a 193.30% increase in the leaf biomass compared to 400 µmol/mol (CO_2_ 400) and 226.34% enhancement than CO_2_ 400, G20. 

Results collected after 6 weeks of growth showed that CO_2_ 800 had the highest leaf dry weight (151.05 ± 0.140 g/m^2^) while CO_2_ 400, G20 had the lowest dry weight (41.52 ± 0.087 g/m^2^). Furthermore, the flavonoid*leaf biomass amount [total flavonoid concentration (mg/g leaf DW) multiplied leaf biomass (g/m^2^)] was significantly different between 400 and 800 µmol/mol CO_2_ concentrations. The CO_2_ 800, G20 in the 4th week had the highest amount of flavonoid*biomass, which was 21.8% higher than CO_2_ 800 in the 3rd week (as its highest level time for TFC; Figure 1).

Other studies have also reported similar results in strawberry plants. Deng and Woodward [18], for example, pointed out that after growing strawberry plants in air containing an extra 170 ppm of CO_2_, the total fresh fruit weights were 42% and 17% higher than weights exhibited by control plants grown at high and low soil nitrogen contents, respectively. Additionally, Bushway and Pritts [19] found that a two- to three-fold enhancement in the air’s CO_2_ content increased strawberry fruit yield by an average of 62%. Furthermore, Campbell and Young [20], Keutgen *et al.* [21], and Bunce [22] highlighted positive strawberry photosynthetic responses with additional 300 ppm CO_2_ ranging from 9% to 197%. Finally, Desjardins *et al.* [23] demonstrated 118% enhancement in photosynthesis in reaction to 600 ppm increase in the air’s CO_2_ concentration.

To date, various methods have been developed and introduced to increase the quality and quantity of plants, and CE provides the technology for *C.asiatica* to respond to CO_2_ enrichment by accelerating the growth rate of the plant including the leaf, producing higher biomass, and decreasing the time to harvest when CO_2_ concentration is increased from 400 to 800 µmol/mol.

The response of these accessions (*C.asiatica*) is consistent with those reported for various food and ornamental plants over the years [5,24,25]. These growth effects are commonly connected with increased rate of leaf photosynthesis when CO2 ceases to be a restrictive factor for carbon assimilation. The experiment by Yu *et al.* [26] specified that exposure to UV-B alone considerably reduced dry weight and photosynthetic rate, while CO_2_ enrichment alone increased dry weight and photosynthetic rate. In their study, they also observed that the dry weight and photosynthetic rate of *P. subcordiformis* grown under the combination of UV-B and CO_2_ had no significant difference compared tothat grown under ambient UV-B and ambient CO_2_.

### 2.2. Leaf Gas Exchange Measurement

For this study, the net photosynthetic rate (PN), stomatal conductance (gs), intercellular CO_2_ concentration (Ci), and transpiration rate (E) were determined by a portable infrared photosynthesis system LI-6400 (LI-COR, Lincoln, NE, USA) from 8:30 am to 10:30 am. During the experiment, photosynthetic photon flux density (PPFD) and leaf temperature were maintained at 1000 μmol/m^2^/s and 30 °C, respectively.

According to Jackson *et al.* [27], water use efficiency is defined as the ratio between the quantities of CO_2_ assimilated by photosynthesis to the [quantity of] water lost through transpiration.

Based on the results gathered in this study, significant differences [in the leaf gas] were found among the four treatments.

**Figure 2:**
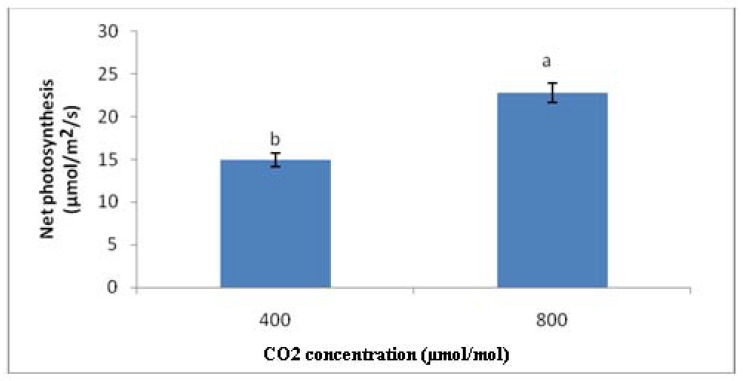
Effects of different CO_2_ concentration on net photosynthesis of irradiated and non-irradiated plants of *C.asiatica*; n = 4 (CO_2_ 400 = Ambient CO_2_; CO_2_ 800 = 800 μmol/mol CO_2_ concentration).

**Figure 3:**
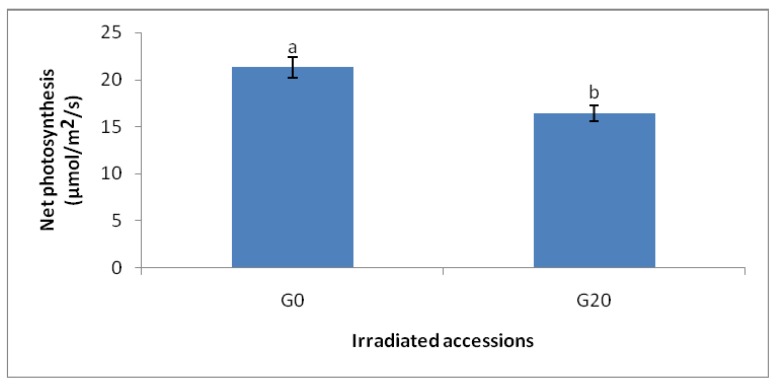
Effects of different gamma irradiation dosage on net photosynthesis of plants of *C.asiatica;* n = 4 (G0 = Control; G20 = Plants irradiated to 20 Gy).

In general, the photosynthesis rate in CO_2_ 800 µmol/mol had significantly the highest value compared to other treatments. All elevated CO_2_ treatments had higher PN than the ambient ones. In other words, CO_2_ 800 and CO_2_ 800, G20 displayed 44.47% and 64.02% higher PN compared to CO_2_ 400 and CO_2_ 400, G20, respectively (Figure 2 and Figure 3).

When comparing the combined effects of high level of CO_2_ (800µmol/mol) and gamma irradiation treatments (20 Gy) with the control plants, it is found that there were significant differences in the photosynthesis rate. More specifically, CO_2_ 800, G20 had 16.95% higher PN than CO_2_ 400, but 23.52% lower than CO_2_ 800. 

**Figure 4:**
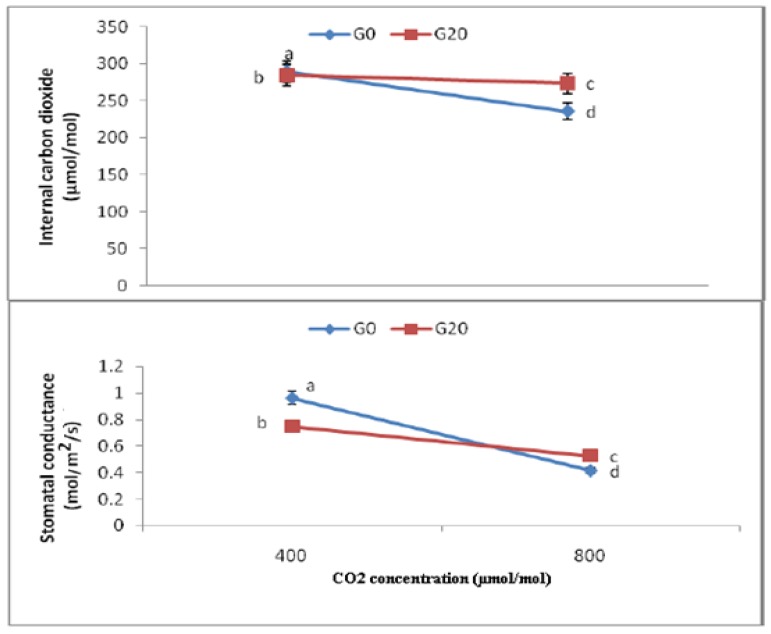
Effects of different CO_2_ concentration on stomatal conductance and internal carbon dioxide of plants of *C.asiatica*; n = 2 (400 = Ambient CO_2_; 800 = 800 μmol/mol CO_2_ concentration).

As shown in Figure 4, when the CO_2_ level was increased from 400 to 800 µmol/mol, stomatal conductance had the tendency to decrease. The lowest gs were detected in CO_2_ 800, which was 56.87% lower than that for CO_2_ 400 as control. Moreover, combined CO_2_ and gamma treatments (CO2 800, G20) resulted in 29.46% lower gs than that for CO_2_ 400, G20, even though CO_2_ 400, G20 itself had 26.89% higher gs than CO_2_ 800.

There were also significant differences among treatments with respect to Ci. In general, it was observed that leaf intercellular carbon dioxide declined significantly with increasing CO_2_ concentration. Specifically, the Ci of CO_2_ 800 declined by 18.56% compared to CO_2_ 400 as control (Figure 4). Moreover, combined CO_2_ and gamma treatments (CO_2_ 800, G20) had 3.86% and 5.52% lower Ci than CO_2_ 400, G20 and CO_2_ 400, respectively, whereas its Ci was 13.8% higher than CO_2_ 800. Meanwhile, the highest Ci was found in CO_2_ 400 (289.5 ± 0.597), while the lowest was found in CO_2_ 800 (235.75 ± 0.790).

**Figure 5:**
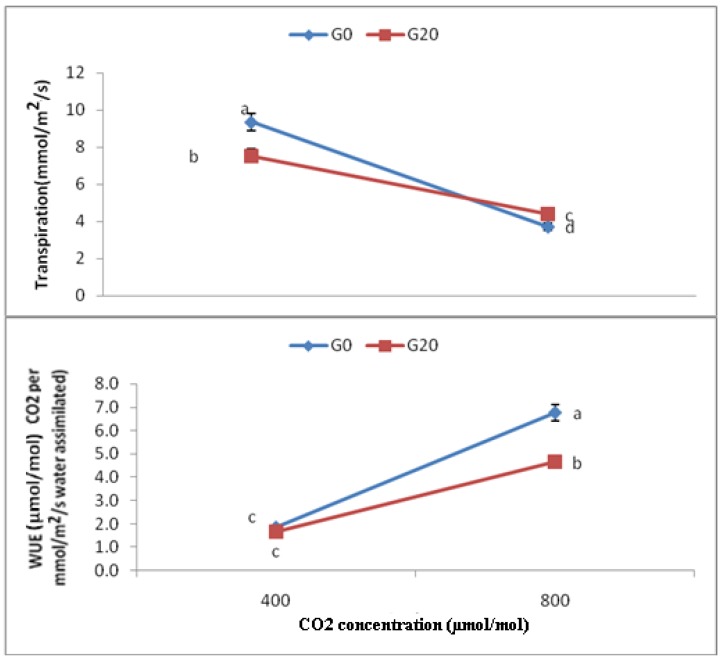
Effects of different CO_2_ concentration on WUE and transpiration of plants of *C. asiatica;* n= 4 (400 = Ambient CO_2_; 800 = 800 μmol/mol CO_2_ concentration).

There were significant differences among treatments in terms of E. In general, the transpiration rate reduced with increasing CO_2_ concentration. Particularly, CO_2_ 800 had 60.18% reduction in E compared to CO_2_ 400 as control (Figure 5). Moreover, combined CO_2_ (800 µmol/mol) and gamma treatment (CO_2_ 800, G20) had 41.89% and 53.21% lower E than those for CO_2_ 400, G20 and CO_2_ 400, respectively, whereas its E (CO_2_ 800, G20) was 17.5% higher than CO_2_ 800. Meanwhile, the highest E was found in CO_2_ 400 (9.36 ± 0.045), while the lowest was found in CO_2_ 800 (3.72 ± 0.082).

Results on WUE treatments of *Centella asiatica* demonstrated significant differences. In general, the water use efficiency increased in response to elevated CO_2_ concentration (Figure 5). More specifically, CO_2_ 800 showed a 263.7% enhancement in E compared to that for CO_2_ 400 as control. Moreover, combined CO_2_ (800 µmol/mol) and gamma treatments (CO_2_ 800, G20) had 182.25% and 150.06% higher WUE than those for CO_2_ 400, G20 and CO_2_ 400 respectively. On the contrary, its WUE (CO_2_ 800, G20) was 45.45% lower than that for CO_2_ 800. Meanwhile, the highest WUE was found in CO_2_ 800 (6.769 ± 0.199), while the lowest was found in CO_2_ 400, G20 (1.648 ± 0.0110) and CO_2_ 400 (1.861 ± 0.0082) (µmol/mol CO_2_ per mmol/m^2^/sec water assimilated).

These findings further support the idea of increased carboxylation activity of ribulose 1,5-bisphosphate carboxylase oxygenase enzyme (Rubisco) in leaves under elevated level of carbon dioxide enhances the net photosynthesis, particularly in C_3_ species. In effect, stomatal conductance will decline, which may result in less transpiration per unit leaf area. According to Morison [28], as the carbon dioxide level doubled, stomatal conductance declined by 30–40%. Nevertheless, this amount varies among species. Concomitantly, water use efficiency (WUE) will increase. The reason for this increase is mainly due to an increase in the net photosynthesis rate than the reduction of water loss through partially closed stomata. Consequently, more dry matter can be made per unit of water used [29,30].

In buckwheat leaves, the PN still declined through higher Ci value, owing to lower gs as a consequence of limited carboxylation efficiency and CO_2_ assimilation, because of the excessive accumulation of free radicals in mesophyll cells by irradiation. [31]. Hence, it indicates declining of Rubisco efficiency, which means higher Ci, always can’t be resulted in higher PN.

On another note, short term exposure to elevated CO_2_ levels is demonstrated to promote net photosynthetic rate in C_3_ plants because the existing ambient CO_2_ concentration is inadequate for Rubisco [32]. An enhancement in CO_2_ accessibility increases carboxylation and reduces the oxygenase activity of Rubisco (which catalyzes either the carboxylation or the oxygenation of ribulose-1 5-bisphosphate with carbon dioxide or oxygen), therefore reducing the CO_2_ loss through photorespiration. Consequently, a net increase in photosynthesis takes place due to the procession of extra CO_2_ [33,34]. An increase in the net photosynthesis in elevated CO_2_ is predicted in spite of whether Rubisco activity or regeneration of ribulose-1, 5-bisphosphate (RubP) is restricting assimilation, and whether the light is saturating or limiting [32].

In another major study, Zobayed *et al.* [35] pointed out that St. John’s wort plants grown under high level of CO_2_ enriched condition (1,500 µmol/mol) displayed increased secondary metabolite production and net photosynthetic rates.

### 2.3. Determination of Lipid Oxidation (Malondialdehyde (MDA))

**Figure 6:**
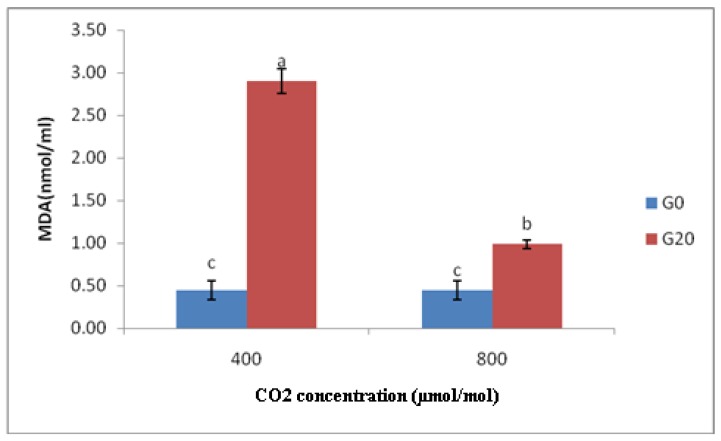
MDA content in different CO_2_ concentration in irradiated and non-irradiated accessions of *C.asiatica*; n = 2 (400 = Ambient CO_2_; 800 = 800 μmol/mol CO_2_ concentration; G0 = Control; G20 = Plants irradiated to 20 Gy).

Changes in the MDA content of *Centella asiatica* accessions under different CO*2* treatments are shown in Figure 6. The MDA contents of *Centella asiatica* accessions exposed to gamma ray and high CO2 concentration (CO_2_ 800, G20) were significantly decreased (65.98%) compared to those that were irradiated, but had ambient CO_2_ level (CO2 400, G20). Yet, no significant difference in MDA content was detected among the non-irradiated plants exposed to different concentrations of CO_2_.

MDA is a product of lipid peroxidation and is commonly used as an indicator in stress physiology of plants. Juan *et al.* [26] reported that MDA content declined in *P. subcordiformis* grown under high levels of CO_2_, indicating that lipid peroxidation was decreased. The fact that UV-B increased MDA content revealed that the membrane was severely damaged. The combination of UV-B and CO_2_ notably reduced MDA content compared to UV-B alone. Additionally, the results of this study also specified that elevated CO_2_ could alleviate UV-B-induced damage to membrane.

Doubling the present atmospheric CO_2_ will modify the CO_2_/O_2_ ratio at Rubisco fixation site, causing 50% reduction in the ratio of photorespiration [36] and about 25%–60% enhancement in the activated state of Rubisco. Moreover, in terms of Juan *et al.* [26] description, the reduction in lipid peroxidation of *P. subcordiformis* at high concentration of CO_2_ might be due to the additional carboxylation reaction of Rubisco, restricted photorespiration and less photoreduction of dioxygen. In this case, fewer electrons were transported to dioxygenduring photosynthesis and in effect, reducing the potential damage of active oxygen to membrane system.

## 3. Experimental

### 3.1. Experimental Design

All experiments involving CO_2_ chamber were designed separately. For flavonoid determination, split split plot based on RCBD were used. The factors were two CO_2_ concentrations (400 and 800 µmol/mol) for two hours, two irradiated and non-irradiated accessions and six leaf samplings (every week). For other parameters, the design was split plot [two CO_2_ concentrations (400 and 800 µmol/mol) and two irradiated and non-irradiated accessions].

### 3.2. Plant Materials and Growth Chamber

The chamber model used to grow *C.asiatica* in this study was a PGW 36 distributed by CONVIRON. It has a microprocessor control system, in which through it, user isable to control temperature, lighting, relative humidity, carbon dioxide and other environmental conditions in the chamber. *C.asiatica* accessions were grown in a chamber to provide the appropriate temperature, relative humidity, and CO_2_ for the irradiated and non-irradiated plants. The photoperiod and light intensity were managed in a controlled environment chamber.

The three plants of *C.asiatica* were planted in individual polybags (40 × 35 cm) filled with sand, coco dust and compost in the ratio of 1:1:1 and organic fertilizer (200 g) per polybag. The ingredients of the organic fertilizer include N (3.5%), P (2.5%), K (2%), Ca (2%), Mg (1%), ZN (0.5%), Bo (0.5%), and Mn (0.5%). The environmental set points for plants were 30 °C, 75% RH, and 300 µmol/m^2^/s PPF with a 14-h light/10-h dark photoperiod. CO_2_ concentrations of 400 and 800 µmol/mol were maintained in the chambers for 2 hours every day between 8.30 to 10.30 am. Irradiation of *Centella asiatica* was conducted in University Kebangsaan Malaysia using Gammacell 220 Excel Irradiator. The source of gamma rays was Cobalt 60.

### 3.3. Plant Harvest (Leaf Biomass)

Growth measurement and plant harvest were conducted once every week. At each harvest, the leaf biomass per plant was determined by oven-drying the leaves at 45–50 °C until it reached a constant mass.

### 3.4. Determination of the Total Flavonoid Content

The flavonoid contents in extracts were determined spectrophotometrically according to the method established by Lamaison and Carnat [37], which is based on the formation of a flavonoid-aluminium coloured characterized by a wavelength with a maximum absorption of 430 nm. After oven drying samples at 45–50 °C, they were kept in a −20 °C freezer. Each sample (1 g) was added to 80% methanol (20 mL) and incubated in the Orbit Shaker at 250 rmp and 50 °C for 2 hours. The samples were then filtered. Extract (1 mg) and 2% AlCl_3_ methanol solution (1 mL, 2 mg AlCl_3_ added to 100 mL absolute methanol) was prepared and kept at room temperature for 15 minutes. Finally, the absorbance was measured at 430 nm using a spectrophotometer. Rutin was used to create the calibration curve and the flavonoids content were expressed in mg per g of rutin equivalent (mg/g).

### 3.5. Gas Exchange

For mature and high flavonoid content leaves, the net photosynthetic rate (PN), stomatal conductance (gs), intercellular CO_2_ concentration (Ci), and transpiration rate (E) were measured in the growth chamber using a portable infrared photosynthesis system LI-6400 (LI-COR, Lincoln, NE, USA) at standard cuvette condition including 1,000 µmol/m^2^/s PPFD, 400 µmol/mol CO_2_ reference, 50–60% relative humidity, and 30 °C leaf temperature. 

### 3.6. Estimation of Lipid Oxidation (Malondialdehyde (MDA))

The level of lipid peroxidation is stated as the content of MDA. The first method to assess the TBA-MDA complex in plant tissue was offered by Heath and Packer [38]:

MDA equivalents (nmol/mL) = [(A532−A600)/155000] × 10^6^

where 532 nm represents the wavelength of maximum absorption of the TBA-MDA complex, A600 is a correction for non-specific turbidity, and 155,000 is the molar extinction coefficient for MDA.

There are possible explanations for the results of the current study which indicate that the synthesis of carbon-rich secondary chemicals was restricted by the accessibility of photosynthates, and that growth processes dominated over differentiation and/or production of defence-related secondary metabolites as long as conditions were favourable for growth. When growth was more restricted than photosynthesis, then the distribution towards defence would increase.

## 4. Conclusions

Returning to the hypothesis posed at the beginning of this study, it is now possible to state that, the results of this study clearly demonstrate the potential of using CO_2_ and gamma to increase the production and quality of *C.asiatica*. This is significant because the proposed method has the potential to increase the product value by reducing the time to harvest, increasing the yield per unit area via boosting photosynthesis capacity as well as increasing biochemicals (flavonoids) per gram DM. 

Collectively, the enhancement in yield and quality provides an economic motivation to produce a consistent pharmaceutical-grade product for commercial purpose. The observations by Estiarte *et al.* [17], who reported higher flavonoid concentration in wheat after CO_2_ enrichment, are in agreement with the results of this study. The present findings also seem to be consistent with the hypothesis that higher carbon accessibility can be invested in flavonoids.

The effect of UV-B irradiance on the growth and flavonols of *Gnaphaliumluteo-album* was studied by Cuadra *et al.* [39]. Their experiments revealed that high-irradiance treatment declined the biomass (leaf weight) and increased flavonols. This is in agreement with the results of this study, in which instead of UV, gamma irradiation was applied to create oxidative stress. In conclusion, several mechanisms can be involved in modifying *C.asiatica* response to CO_2_ enrichment by applying gamma radiation. For example, a damaged photosynthetic apparatus under enhanced gamma or UV-B may alter the photosynthetic response to elevated CO_2_ [40].

The following conclusions can be drawn from the present study, that elevated CO_2_ could reduce the MDA content induced by gamma radiation, and therefore reducing the oxidative damage to *C. asiatica*. The most important cause for lower oxidative stress in *C.asiatica* grown under elevated CO_2_ lies in the fact that high concentration of CO_2_ possibly enhances the ratio of CO_2_/O_2_, increases the assimilation of CO_2_, declines the formation of ROS (Reactive oxygen species) as the product of O_2_ acting as electron receptor, and finally reduces the formation of H_2_O_2_ as the product of photorespiration, thereby reducing MDA.

This result may be explained by the fact that *C.asiatica* grown under high CO_2_ is better in overcoming the detrimental impact of stress factor (gamma radiation) that acts via the generation of activated oxygen species. The explanation of the results for *C.asiatica* grown under the combination of elevated CO_2_ and gamma are complex. There are, however, possible interpretations. Enhanced gamma and CO_2_ concomitantly and synergistically resulted in boosting flavonoid concentration, and protecting and ameliorating the photosynthesis system. Although, CO_2_ enrichment compensated the adverse effects of gamma, the plant also benefits from gamma irradiation to significantly boost flavonoids components.

These findings provide the following insights for future research on *C.asiatica* such as using gamma greenhouse, gamma field or ion beam supplemented with CO_2_ in different types of settings such as an open top chamber, as well as applying CO_2_ in a bioreactor, which remain to be done in the future.
